# Anti-Obesity Effects of Traditional and Commercial Kochujang in Overweight and Obese Adults: A Randomized Controlled Trial

**DOI:** 10.3390/nu14142783

**Published:** 2022-07-06

**Authors:** A Lum Han, Su-Ji Jeong, Myeong-Seon Ryu, Hee-Jong Yang, Do-Youn Jeong, Do-Sim Park, Hee Kyung Lee

**Affiliations:** 1 Department of Family Medicine, Wonkwang University Hospital, Iksan 54538, Korea; yebbnkim@gmail.com; 2 Microbial Institute for Fermentation Industry, Sunchang 56048, Korea; yo217@naver.com (S.-J.J.); rms6223@naver.com (M.-S.R.); godfiltss@naver.com (H.-J.Y.); jdy2534@korea.kr (D.-Y.J.); 3 Department of Laboratory Medicine, Wonkwang University Hospital, Iksan 54538, Korea; emailds@hanmail.net

**Keywords:** capsaicin, phytochemicals, fermentation, anti-obesity, kochujang

## Abstract

Kochujang shows anti-obesity effects in cell and animal models. Kochujang is traditionally prepared via slow fermentation or commercially using *Aspergillus oryzae*. We analyze the anti-obesity effects of two types of Kochujang in overweight and obese adults. The analyses included the following groups: traditional Kochujang containing either a high-dose (HTK; *n* = 19), or a low-dose of beneficial microbes (LTK; *n* = 18), and commercial Kochujang (CK; *n* = 17). Waist circumference decreased significantly in the HTK and CK groups. Total cholesterol, low-density lipoprotein cholesterol, high-density lipoprotein cholesterol, and triglyceride levels decreased in the HTK and LTK groups. Visceral fat is significantly reduced in the HTK group. The population of beneficial microorganisms in stool samples increased in all groups. Consumption of Kochujang reduces visceral fat content and improves the lipid profile, which can be enhanced by enrichment with beneficial microbes. These results suggest that Kochujang has the potential for application in obesity prevention.

## 1. Introduction

The prevalence of overweight and obesity worldwide is increasing, with nearly one-third of the world’s population now classified as overweight or obese [1]. Obesity adversely affects almost every function of the body, contributing to several disease states such as diabetes, cardiovascular disease, several types of cancer, various musculoskeletal disorders, and poor mental health [1]. While obesity is on the rise worldwide, the anti-obesity effect of Kochujang is attracting attention. Kochujang is one of Korea’s representative sauces with a combination of flavors. Kochujang has a savory taste owing to proteins derived from soybeans; sweetness from carbohydrates obtained from glutinous rice, non-glutinous rice, and barley rice; a spicy taste from red pepper powder; asalty taste from salt used for seasoning [2]. The fermentation process of food is associated with several health benefits, such as the degradation of toxic substances, improvement of digestion, and the production of vitamins that increase the nutritional value [3]. The weight loss- and obesity-reducing effects of Kochujang have been reported in several studies. Kochujang contains approximately 20–30% red pepper powder, rich in capsaicin, which exhibits anti-obesity effects [4]. The capsaicin present in red pepper powder can improve energy metabolism, lower cholesterol levels, improve blood lipid profiles, and break down fat tissues [5]. The use of red pepper powder has been shown to alter thermogenesis and appetite [6]. Other studies have reported changes in appetite and energy balance upon ingestion of capsaicin [7,8]. This change may occur via changes in the hypothalamic anorexia and anorexia neuropeptides [9]. Meju, the main component of Kochujang, is a fermented soybean product that shows anti-obesity and anti-atherosclerotic activities in obese adults [10]. Kochujang can be prepared using traditional methods or via commercial mass production. Generally, traditional Kochujang is prepared by fermenting Meju, salt, and red pepper powder together with glutinous rice and malt [11]. In contrast, for commercial Kochujang preparation, koji products are manufactured by culturing fungi such as *Aspergillus oryzae* on rice grains or flakes [11]. As all of the main ingredients of Kochujang have anti-obesity effects, Kochujang itself may show anti-obesity effects. However, to date, clinical trials comparing the post-use effects of traditional and commercial Kochujang in human participants are lacking. In this randomized, double-blind clinical trial, we tested the hypothesis that traditional and commercial Kochujang supplementation can reduce body fat content and improve blood lipid profiles in overweight adults. Further, the difference in anti-obesity effects between traditional and commercial Kochujang was investigated. The effects of traditional and commercial Kochujang supplementation on microbial changes in the stool were also evaluated.

## 2. Materials and Methods

### 2.1. Study Design

This study was a 6-week randomized, double-blind clinical trial (registration number: KCT0006899). The participants were asked to visit the research center three times. After 3 weeks of Kochujang supplementation, the participants visited the research center to check vital signs/side effects, and adherence to dosing. Efficacy indicators such as body weight, abdominal fat computed tomography (CT), adipokine levels, lipid profiles, and blood tests were evaluated at the first and last visits. The following Kochujang pills were provided to the participants: a pill prepared using traditional Kochujang with a high content of beneficial microorganisms (HTK), a pill prepared using traditional Kochujang containing a low content of beneficial microorganisms (LTK), and a pill prepared using commercial Kochujang (CK). Enrolled participants were to be randomly assigned to one of three groups receiving HTK pills (*n* = 20), LTK pills (*n* = 20), or CK pills (*n* = 20). The number of screened participants was 62 and the dropout rate was 20%. In total, 48 participants (16 participants in each intake group) completed the study.

**Figure 1:**
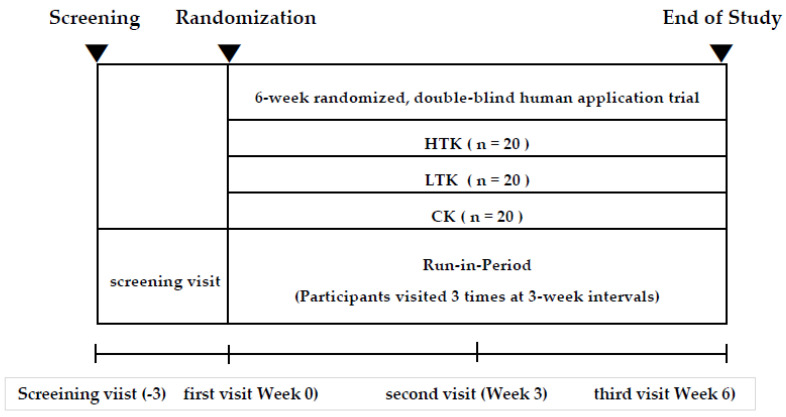
Study design.

The following method was used to conduct the double-blind randomized trial: a screening number was provided to participants who provided written consent to participate in the study. The screening number consisted of a total of four digits, beginning with S followed by three digits (for example, S001). Participants who passed the screening test were assigned a study subject number. The study subject number was randomly assigned in order. The study subject number began with GCJ-R and had a set rule with the last two digits. The numbers indicated the order of participants in the study and ranged from 01 to 62. The subject number assigned to each subject was used as a subject identification code to identify the subject until the end of the trial. Participants were asked to continue their normal diet and not consume other nutraceuticals or dietary supplements. Nutrient intake levels and activity levels were measured before and after the intervention period. At the beginning of the trial, all participants were instructed to maintain a usual diet and level of physical activity. The study design is presented in Figure 1.

### 2.2. Participants

In total, 62 volunteers aged 19–70 years with a body mass index (BMI) of ≥23 kg/m^2^ were recruited and randomly assigned to three groups. Exclusion criteria were as follows: >10% change in body weight in the past 3 months; cardiovascular diseases such as arrhythmia, heart failure, myocardial infarction, and use of a pacemaker; allergy or hypersensitivity to any component of the test product; colon diseases such as Crohn’s disease; history of gastrointestinal surgery (such as appendix or intestinal surgery); participation in another clinical trial within the past 2 months; liver dysfunction or acute/chronic renal disease; use of antipsychotic drug therapy within the past 2 months; laboratory test abnormalities as assessed by the investigator; psychological state abnormalities; history of alcohol or drug abuse; pregnancy or breastfeeding. All 62 volunteers provided consent prior to treatment. Participants under treatment with additional medicines for personal conditions during Kochujang supplementation and those who intended to stop Kochujang supplementation were excluded. The protocol was approved by the institutional review board of Wonkwang University Hospital (IRB approval no. 2021-04-046).

Participants consumed Kochujang pills thrice a day after every meal, and the total amount of Kochujang pills taken per day was 25.3 g (19 g/d as Kochujang powder). In the 2014 National Health and Nutrition Survey, the average daily intake of red pepper paste for Koreans was 10.75 g and the extreme intake was 3686 g [12]. Therefore, in this study, 19 g of Kochujang powder was set as the daily intake.

### 2.3. Preparation of Kochujang Products

**Figure 2:**
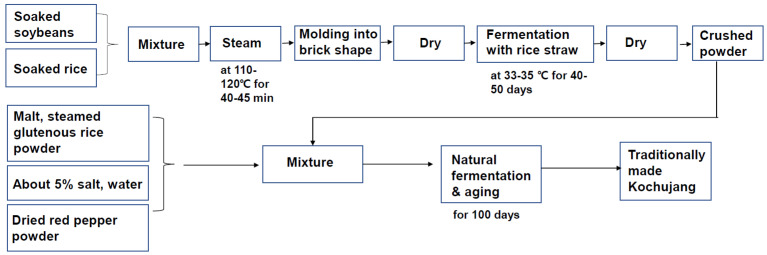
Schematic representation of the traditional process for Kochujang preparation.

The preparation of Kochujang varies with region and household. Generally, Kochujang is prepared by mixing ingredients such as glutinous rice, red pepper powder, malt, and soybean paste powder in the fermentation process. Meju, which is one of the major ingredients, is prepared between August and September for fermentation prior to cold weather. Generally, Kochujang is prepared during the cold season between October and March. Prepared Kochujang can be consumed immediately; however, it is allowed to ripen for a few months for improved quality and flavor. The preparation process of traditional Kochujang is shown in Figure 2.

Kochujang in pill form was prepared by mixing the Kochujang sample (Microbial Institute for Fermentation Industry, Sunchang, Korea) with deionized water to reduce the influence of high sugar content on removing moisture, and then the sample was freeze-dried. Dried samples were ground and then mixed with microcrystalline cellulose and magnesium stearate (Microbial Institute for Fermentation Industry) in defined proportions as shown in Table 1.

### 2.4. Safety Assessment

The health of the participants was assessed via screening tests, including electrocardiogram, urinalysis, hematology, and blood chemistry tests. These included analyses of white and red blood cell counts; hemoglobin, hematocrit, and platelet counts; total protein, albumin, alanine aminotransferase (ALT), aspartate aminotransferase (AST), blood urea nitrogen (BUN), and creatinine levels. Pulse and blood pressure were measured at each visit after a 10-min break. Participants were asked to report any side effects or changes in lifestyle or eating patterns during Kochujang supplementation and to report medication compliance.

### 2.5. Biochemical Analyses, Abdomen Fat CT Analysis, and Lifestyle Survey

A dietary intake survey was performed based on the Meal Recording Act; the research participants received a dietary log and recorded all food they consumed as accurately as possible [13]. Research participants completed a physical activity survey questionnaire based on the Global Physical Activity Questionnaire during their visits [14]. To analyze changes in the intestinal microbiome, ≥1 g feces was frozen using a stool collection kit after the first visit and 6 weeks later. The stool samples before and after Kochujang supplementation were collected from each subject using MICROBE & ME stool collecting kit (Macrogen, Seoul, South Korea). For CT scanning of abdominal fat, CT scans (Hispeed CT/e; General Electric, Boston, MA, USA) were taken with participants in the supine position. Total abdominal fat area, subcutaneous fat area, and visceral fat area were measured and expressed as mm2. For blood tests, the participants fasted for >12 h before blood collection. The total cholesterol (TC), low-density lipoprotein cholesterol (LDL-C), high-density lipoprotein cholesterol (HDL-C), triglyceride (TG), non-HDL-cholesterol (NonHDL-C), ALT, AST, gamma-glutamyl transferase, BUN, creatinine, glucose, and high sensitivity C-reactive protein levels were measured using the Hitachi 7600 automatic analyzer (Hitachi, Tokyo, Japan). Serum leptin and plasma adiponectin levels were measured via radioimmunoassay using a human leptin kit (Linco Research, St. Charles, MO, USA).

### 2.6. Statistical Analyses

All statistical analyses were performed using PASW statistics 23 (previously SPSS statistics) (SPSS version 23.0; IMP SPSS, Chicago, IL, USA). All data are expressed as the mean ± standard error or percentages (%) for categorical variables. Values of *p* < 0.05 were considered significant.

The sample size was determined to achieve 80% statistical power with an alpha of 0.05. The sample size for each group was determined by allowing a dropout rate of 20%. Efficacy parameters were analyzed in the per-protocol group, and safety parameters were analyzed in the intention-to-treat group. A chi-square test was performed to determine baseline differences in the frequencies of categorized variables between groups. Student’s paired *t*-test was performed to assess differences between groups before and after the 6-week intervention period. A linear mixed-effects model was applied to the repeated measures data for each continuous outcome variable and data. An expert analyzed the 24-h dietary intake data using Can-Pro 3.0 software (Korean Nutrition Society, Seoul, South Korea).

## 3. Results

### 3.1. Participants

A total of 62 participants were recruited; four were excluded during the screening process, two did not meet the inclusion criteria, and two declined participation. The remaining 58 patients were randomly assigned to the following three groups: HTK, LTK, and CK. All participants completed the 6-week study, except for four who withdrew consent. The final analysis included a total of 54 participants in the HTK (*n* = 19), LTK (*n* = 18), and CK (*n* = 17) groups (Figure 1). Four individuals dropped out, two withdrew consent, one required treatment for a hand fracture, and one required treatment for dermatosis. This was not related to Kochujang supplementation, and none of the participants complained of side effects.

### 3.2. Anthropometric Parameters

The general characteristics of the participants are shown in Table 2. Cross-analysis was performed for sex, alcohol consumption, and smoking; analysis of variance was performed for age, weight, and BMI. There were no significant differences in baseline characteristics among the three groups, such as age, sex, weight, height, alcohol consumption, smoking, initial weight, and initial BMI. No significant changes in caloric intake were found within or between groups in the dietary intake survey (*p* > 0.05). No significant changes in the metabolic equivalent of the task were found within or between groups in the physical activity survey (*p* > 0.05).

### 3.3. Efficacy Evaluation

**Figure 3:**
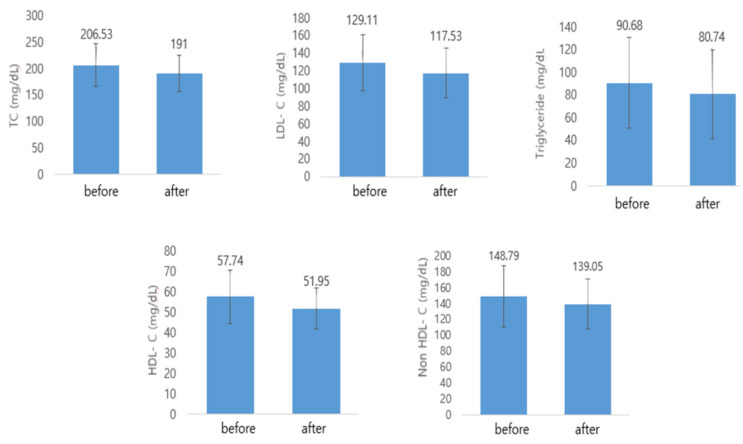
Lipid profile before and after Kochujang treatment in the HTK group. HTK, traditional Kochujang containing a high dose of beneficial microbes. TC, total cholesterol; LDL-C, low-density lipoprotein cholesterol; HDL-C, high-density lipoprotein cholesterol; NonHDL-C, non-HDL-C.

**Figure 4:**
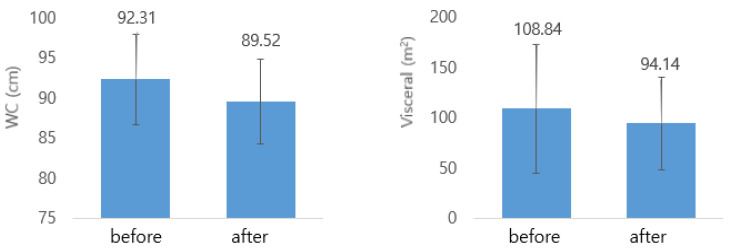
WC and visceral fat area before and after Kochujang treatment in the HTK group. HTK, traditional Kochujang containing a high dose of beneficial microbes; WC, waist circumference.

Waist circumference (WC) decreased significantly after Kochujang supplementation in the HTK and CK groups. Hip circumference (HC) and waist-hip ratio (WHR) decreased in all three groups; however, the reduction was only significant in the CK group. TC, LDL-C, HDL-C, TG, and NonHDL-C levels decreased in the HTK and LTK groups; however, the reduction was significant in only the HTK group (Figure 3). Visceral fat area, subcutaneous fat area, and visceral fat/subcutaneous fat ratio (V/S) decreased in the HTK and LTK groups; however, the decrease in the visceral fat area was significant only in the HTK group (Figure 4). In all three groups, there was no significant change in BMI, body fat mass (BFM), or percent of body fat (PBF) before and after treatment (Table 3).

### 3.4. Microbiome Analysis

The presence of beneficial, harmful, and other microorganisms was evaluated in stool samples. After Kochujang supplementation, the population of beneficial microorganisms increased in all three groups, and that of harmful microorganisms decreased in the HTK and CK groups; however, this was not significant. Beneficial microorganisms included *Lactobacillus* spp., *Bifidobacterium* spp., *Lactococcus lactis*, *Enterococcus faecium*, and Bacteroides. Harmful bacteria include *Clostridium perfringens*, *Bacteroides eggerthii*, *Sutterella stercoricanis*, *Ruminococcus torques*, *Parabacteroides merdae*, and *Parabacteroides distasonis* (Table 4).

## 4. Discussion

Epidemiological studies have shown that the incidence of obesity is increasing worldwide and in Korea [15]. Fermented foods have been suggested to serve as a source of healthy nutrients such as dietary fiber, antioxidants, phytochemicals, and minerals [16]. Among various fermented products, Kochujang is a traditional Korean sauce that can be used to prepare various food products or consumed for its health benefits [17]. The anti-obesity effect of Kochujang has been proven in several animal experiments [4,18]. In the present study, we presented evidence that Kochujang consumption may reduce visceral fat content and improve blood lipid profiles in overweight and obese patients. We also found that the population of beneficial bacteria may increase in the stool microbiome upon Kochujang supplementation.

Kochujang can be prepared via two approaches. One approach involves the fermentation of soybean paste (fermented soybean), glutinous rice, and red pepper powder by bacteria (Bacillus subtilis and *Bacillus licheniformis*) or yeast (*Saccharomyces cerevisiae* and *Zygosaccharomyces rouxii*) [19]. This traditional homemade Kochujang preparation method requires at least 6 months of fermentation [19]. The quality of Meju added to Kochujang greatly depends on the microorganisms used in the fermentation process [19]. The second method of Kochujang preparation involves the commercial method, in which steamed rice inoculated with yeast (*A. oryzae*) is used. One of the advantages of using koji is the short period required for fermentation, which usually ranges from 2 weeks to 1 month [19].

Traditional Kochujang contains more carbohydrate sources than those present in other traditional sauces and may contain a large population of various bacteria [20]. Since the presence of beneficial bacteria such as *Bacillus* spp. in Kochujang varies with fermentation conditions and component composition [20], the health effects may differ between traditional Kochujang containing varying amounts of microorganisms and commercial Kochujang. Therefore, we divided the traditional Kochujang administration group into two groups and used Kochujang inoculated with a high content of an effective strain and Kochujang inoculated with a low content. The anti-obesity effects of two types of traditional Kochujang and commercially available Kochujang were compared.

A previous study investigated the anti-obesity effect of Kochujang according to the polymorphism of the obesity-related gene, peroxisome proliferator activator receptor c (PPARc2) in overweight/obese subjects [21]. Study participants with BMI ≥ 23 or a high waist/hip ratio (WHR; ≥0.90 for men and ≥0.85 for women) received a placebo or Kochujang for 12 weeks. The result showed that neither Kochujang nor placebo had any effect on anthropometric measures such as BMI, WC, BP, VF area, and SF area after the intervention [21]. However, in our study, WC and VF areas decreased after Kochujang treatment with a high content of effective microorganisms added. This may be a result of the increase in the anti-obesity effect of the high-content effective microorganism.

Traditional Kochujang and commercial Kochujang reduced weight gain in a dietary intake experiment with rats [22]. The study investigated the effect of Kochujang on the reduction of body weight, adipose tissue, and serum lipid levels in male Sprague-Dawley rats fed a high-fat diet containing 10% of unfermented traditional Kochujang, fermented traditional Kochujang, or fermented commercial Kochujang. The changes in lipid profile, body weight, and weight of each organ and adipose tissue were measured. The results showed that Kochujang had an anti-obesity effect; however, the non-fermented Kochujang group had no significant effect on organ fat reduction, weight loss, cholesterol, or triglycerides [22].

Perirenal fat and hepatic total lipids were decreased in Kochujang-fed rats. In a human study with overweight participants, the group that was fed Kochujang for 12 weeks showed no change in body weight and WHR compared to the group that was fed the placebo; however, they did show a significant decrease in visceral fat. In addition, serum concentrations of triglycerides and apolipoprotein B were also decreased in the Kochujang-fed group compared to that of the placebo group [23]. In rats fed a high-fat diet, Kochujang supplementation reduces the increase in body fat content by 30%, which has been found to be mediated via an increase in energy expenditure and activity of brown adipocytes [24]. In a similar rat experiment, it was reported that the addition of capsaicin has an anti-obesity effect that reduces the increase in body fat content by 30%, which is attributed to an increase in mitochondrial activity and beta-adrenergic activation of brown adipocytes [25]. However, they reported that capsaicin supplementation does not improve cholesterol, TGL, and HDL-C profiles [25]. The increase in energy expenditure induced by capsaicin occurs via the secretion of catecholamine from the adrenal medulla [26]. Substance P acts as a cooperating factor in the cholinergic regulation of adrenal catecholamine release [26].

Fermentation improves the metabolic function of active ingredients by converting inactive forms present in food to active mediators [27]. It has been reported that daidzein and genistein levels are considerably increased upon fermentation of soybeans [27]. The increased daidzein and genistein content in fermented soybeans is attributed to the contribution of microbial enzymes to hydrolysis during fermentation [28]. During sufficient fermentation, various enzymes are produced by yeast and lactic acid bacteria [28].

Traditional Kochujang composition varies with the province of Korea in production, carbohydrate content, and bacteria present in the fermentation environment [20]. Since Kochujang has a lower salinity and higher carbohydrate content than soybean paste, the growth of harmful bacteria may increase [29]. Traditional Kochujang of high quality should be well managed and contain a large number of beneficial bacteria and <0.5% harmful bacteria [29]. Fecal transplantation studies provide convincing evidence for an association between the microbiome and blood lipids. Indeed, many metabolic phenotypes, including lipid metabolism, obesity, glucose response, and insulin sensitivity, can be transmitted to the host via fecal transplantation. Metagenomic sequencing (MGS) profiling yielded an average of 3.1 Gb of sequencing data for each sample, aligned to 18.6 million bacterial genes. Different microorganisms have varying effects on lipids, further demonstrating that triglycerides with different sized fatty acid moieties are associated with different microorganisms [30]. Although the exact mechanisms of microbiome types and human lipid metabolism are still being studied, they show potential as individualized therapeutics in the future.

The inoculation of Kochujang with beneficial bacteria ensures that the bacterial population is controlled and has specific functions. Kochujang inoculated with *Bacillus* spp. has anti-cerebrovascular disease effects, and the properties differ depending on the province of Korea it is prepared in [20]. Traditional Kochujang has also been found to show a difference in functionality depending on the abundance of microorganisms [20].

We found that Kochujang supplementation had anti-obesity effects. The visceral fat content decreased only in the HTK group. A previous study evaluated the abdominal fat CT scans and blood lipid profiles after administering Kochujang to overweight Korean adults for 12 weeks. They found that although the body weight and WHR did not change, there was a significant reduction in the visceral fat area [23]. In addition, serum TG and apolipoprotein B levels decreased in the Kochujang-administered group compared with those in the control group [23]. These results can be attributed to bioactive compounds such as isoflavone aglycones, peptides in fermented soy, and capsaicin present in red pepper powder via the activity of the obesity-linked gene and peroxisome proliferator activator receptor γ (PPARγ2) [21].

Adiponectin and leptin are adipokines that considerably influence obesity-related metabolic diseases by modulating fat metabolism, energy homeostasis, and insulin sensitivity. Adiponectin and leptin levels are inversely related to the progression of obesity, insulin resistance, and atherosclerosis [31]. In our study, there was no significant change in the levels of leptin and adiponectin in any of the three groups. Previously, leptin secretion was measured upon adding Kochujang and garlic-added Kochujang to cultured 3T3-L1 adipocytes [32]; leptin secretion was found to decrease by 34% and 48%, respectively, compared with that in control adipocytes. mRNA expression levels of obesity-related genes such as tumor necrosis factor-alpha, PPARγ, CCAAT/enhancer-binding protein alpha, and sterol regulatory element-binding transcription factor 1 (SREBP1c) have also been found to decrease in cells treated with Kochujang and garlic-added Kochujang compared with those in the control group [32]. In another study, when 3T3-L1 adipocytes were treated with Kochujang extract, adipocyte size and leptin secretion were found to decrease [33]. The study reported that Kochujang suppresses lipogenesis via downregulation of obesity-related genes SREBP-1c and PPAR-quin and stimulates lipolysis due to increased hormone-sensitive lipase activity [33]. Among healthy Korean participants, a study found that the adiponectin level and risk of metabolic syndrome were reduced with an increase in kimchi consumption [34].

Owing to the clinical nature of this study, it cannot provide information on the exact mechanism of the chemical compounds and their interactions responsible for the biological activity of Kochujang. However, based on the results of previous studies, the anti-obesity effect of Kochujang, which contains a high number of beneficial microorganisms, may be due to the transformation of components such as lipolytic enzymes, certain end-products of Kochujang, and capsaicin into a more active form or some combination of these. Additionally, isomaltooligosaccharide (0.8~6.5%) and small peptides are produced, which have a beneficial effect on fat metabolism [33].

Kochujang ingestion can cause digestive symptoms such as heartburn. Some studies suggest that capsaicin, one of its components, may increase the risk of gastric adenocarcinoma in mice. However, there are studies showing that after adjusting for related factors, there is no relationship with gastric adenocarcinoma; thus, there is no evidence of any negative effects of Kochujang at this point [35].

A major limitation of our study is the small sample size, which limits the generalizability of our results to other populations of overweight or obese individuals. Additionally, changes in certain obesity-related indicators were not significant. During the course of the trial, the food intake and activity levels of the participants were not controlled because we relied on self-reported data.

## 5. Conclusions

In conclusion, the anti-obesity effect of traditional Kochujang may be greater than that of commercial Kochujang, which can be further increased with an increased number of beneficial bacteria present. Traditional Kochujang incorporated into the diet can prevent obesity. Despite the limitations, our study is meaningful in that it observed the anti-obesity effect of Kochujang in human participants with respect to various indicators. The study also compared the number of beneficial bacteria between traditional and commercial Kochujang. Future studies should address the above limitations.

## Figures and Tables

**Table 1:** Composition of Kochujang pills.

Ingredient	HTK		LTK		CK	
	Content (g)	Ratio (%)	Content (g)	Ratio (%)	Content (g)	Ratio (%)
Freeze-dried Kochujang powder	19.0	75	19.0	75	19.0	75
Microcrystalline cellulose	5.1	20	5.1	20	5.1	20
Magnesium stearate	1.2	5	1.2	5	1.2	5
Total	25.3	100	25.3	100	25.3	100

HTK, traditional Kochujang containing a high-dose of beneficial microbes; LTK, traditional Kochujang containing a low-dose of effective microbes; CK, commercially prepared Kochujang.

**Table 2:** General characteristics of participants.

Value	Group			
	HTK (*n* = 19)	LTK (*n* = 18)	CK (*n* = 17)	*p*
Sex (M/F)	6/13	9/11	5/14	0.447
Alcohol consumption (*n*)	11 (57.9)	10 (50.0)	8 (42.1)	0.623
Smoking (*n*)	1 (5.3)	3 (15.0)	1 (5.3)	0.455
Age (years)	41.58 ± 9.19	41.1 ± 10.08	37.16 ± 11.21	0.345
Weight (kg)	70.99 ± 10.21	71.05 ± 9.16	71.44 ± 12.58	0.991
BMI (kg/m^2^)	26.02 ± 2.32	25.87 ± 2.08	26.03 ± 2.71	0.975

The HTK, traditional Kochujang containing a high-dose of beneficial microbes; LTK, traditional Kochujang containing a low-dose of effective microbes; CK, commercially prepared Kochujang; M, male; F, female; BMI, body mass index. Values are presented as the mean ± standard deviation or number (percentage). A significant difference between three groups, *p*-value for independent *t*-test. A significant difference between three groups, *p*-value for independent Chi-square test.

**Table 3:** Efficacy comparison between the three groups.

Value	Group								
	HTK (*n* = 19)			LTK (*n* = 18)			CK (*n* = 17)		
	Before	After	*p*	Before	After	*p*	Before	After	*p*
HC (cm)	101.46 ± 5.64	100.5 ± 4.99	0.099	100.84 ± 4.64	99.48 ± 4.01	0.057	101.66 ± 6.88	100.38 ± 6.73	0.022
WHR	0.91 ± 0.03	0.89 ± 0.03	0.053	0.91 ± 0.04	0.91 ± 0.03	0.956	0.91 ± 0.05	0.89 ± 0.05	0.024
TC (mg/dL)	206.53 ± 39.91	191.00 ± 34.95	0.011	219.06 ± 33.73	218.56 ± 38.31	0.901	205.59 ± 35.21	205 ± 36.92	0.928
LDL-C (mg/dL)	129.11 ± 32.07	117.53 ± 28.50	0.020	138.94 ± 31.16	138.44 ± 35.68	0.912	123.24 ± 30.51	124.41 ± 32.07	0.822
HDL-C (mg/dL)	57.74 ± 13.12	51.95 ± 10.10	0.003	56.28 ± 13.23	55.17 ± 14.87	0.420	62.29 ± 12.39	60.24 ± 9.93	0.152
Triglyceride (mg/dL)	90.68 ± 40.19	80.74 ± 39.20	0.003	118.89 ± 58.96	131.5 ± 60.78	0.389	99.35 ± 78.48	95.47 ± 100.43	0.638
Non HDL-C (mg/dL)	148.79 ± 38.40	139.05 ± 31.44	0.050	162.78 ± 32.57	163.39 ± 37.34	0.869	143.29 ± 33.75	144.76 ± 37.88	0.796
WC (cm)	92.31 ± 5.66	89.52 ± 5.33	0.006	91.94 ± 5.67	90.76 ± 5.56	0.248	92.34 ± 9.21	89.19 ± 8.58	0.002
Adiponectin (μg/mL)	21.48 ± 7.12	20.75 ± 5.53	0.497	20.71 ± 10.84	23.22 ± 11.93	0.099	29.69 ± 10.44	27.59 ± 14.22	0.367
Leptin (ng/mL)	17,109.06 ± 14,015.12	20,527 ± 20,871.21	0.286	12,258.87 ± 10,118.63	15,728.07 ± 8290.3	0.141	23,299 ± 25,617.05	21,998.07 ± 21,734.74	0.764
VF (m^2^)	108.84 ± 63.81	94.14 ± 46.29	0.021	122.18 ± 61.54	119.15 ± 63.47	0.272	90.79 ± 60.55	94.95 ± 58.19	0.086
SF (m^2)^	202.66 ± 64.94	204.32 ± 57.27	0.789	184.94 ± 57.09	182.43 ± 60.39	0.520	218.91 ± 101.03	219.41 ± 83.6	0.949
V/S	0.58 ± 0.48	0.54 ± 0.37	0.403	0.69 ± 0.36	0.69 ± 0.39	0.894	0.45 ± 0.29	0.46 ± 0.28	0.732
GGT (IU/L)	22.11 ± 14.9	21.42 ± 10.95	0.682	25.78 ± 17.45	26.5 ± 19.05	0.622	22.18 ± 18.56	21.76 ± 19.24	0.744
AST (IU/L)	22.05 ± 5.3	22.74 ± 4.85	0.495	22.78 ± 7.97	24.44 ± 10	0.182	20.59 ± 6.96	22 ± 7.09	0.148
ALT (IU/L)	20.79 ± 8.69	21.11 ± 7.79	0.811	27.61 ± 23.86	28.67 ± 28.73	0.668	23.18 ± 14.69	23 ± 17.48	0.928
BUN (mg/dL)	12.63 ± 3.00	13.68 ± 3.43	0.030	13.67 ± 4.7	13.17 ± 4.41	0.480	12.41 ± 3.22	12.18 ± 4.45	0.797
Cr (mg/dL)	0.75 ± 0.17	0.77 ± 0.18	0.108	0.77 ± 0.17	0.78 ± 0.18	0.472	0.71 ± 0.14	0.71 ± 0.16	0.921
Glucose (mg/dL)	105.68 ± 10.59	103.42 ± 8.01	0.250	106.28 ± 10.85	103.89 ± 10.35	0.112	103.94 ± 8.22	101.12 ± 9.77	0.185
hs-CRP (mg/dL)	1.76 ± 3.69	0.80 ± 0.42	0.255	2.59 ± 5.91	1.3 ± 1.16	0.362	1.32 ± 1.93	0.94 ± 0.73	0.421
BMI (kg/m^2^)	26.02 ± 2.32	26.20 ± 2.43	0.201	25.87 ± 2.08	25.97 ± 2.1	0.462	26.03 ± 2.71	26.25 ± 2.63	0.127
Weight (kg)	70.99 ± 10.21	71.44 ± 10.24	0.228	71.05 ± 9.16	71.26 ± 8.94	0.564	71.44 ± 12.58	72.09 ± 12.56	0.095
BFM (kg)	22.74 ± 5.02	23.29 ± 4.79	0.105	23.14 ± 5.04	23.23 ± 5.06	0.798	24.2 ± 8.17	24.52 ± 7.92	0.201
PBF (%)	32.31 ± 6.82	32.87 ± 6.48	0.118	32.76 ± 6.73	32.73 ± 6.65	0.933	33.83 ± 9.14	34.02 ± 9.04	0.460

HTK, traditional Kochujang containing a high-dose of beneficial microbes; LTK, traditional Kochujang containing a low-dose of effective microbes; CK, commercially prepared Kochujang; HC, Hip circumference; WC, waist circumference; BMI, body mass index, WHR, waist-hip ratio; TC, total cholesterol, LDL-C, low-density lipoprotein cholesterol; HDL-C, high-density lipoprotein cholesterol; TG, triglyceride, NonHDL-C, NonHDL-cholesterol; VF, visceral fat; SF, subcutaneous fat; V/S, visceral fat/subcutaneous fat ratio; GGT, gamma-GT; AST, aspartate transaminase; ALT, alanine transaminase; BUN, blood urea nitrogen; Cr, creatinine, glucose, hs-CRP, high-sensitivity C-reactive protein; BFM, body fat mass; PBF, percent of body fat. Values are presented as the mean ± standard deviation. A paired *t*-test was performed to analyze the statistical significance of each variable before and after administration of Kochujang.

**Table 4:** Comparison of microbiome changes in stools between three groups.

Value	Group								
	HTK (*n* = 19)			LTK (*n* = 18)			CK (*n* = 17)		
	Before (%)	After (%)	*p*	Before (%)	After (%)	*p*	Before (%)	After (%)	*p*
Beneficial Bacteria	26.82 ± 11.26	31.04 ± 11.73	0.128	25.61 ± 6.53	29.79 ± 11.44	0.206	27.59 ± 11.72	28.05 ± 9.03	0.898
Harmful Bacteria	5.03 ± 10.01	4.11 ± 7.95	0.157	3.00 ± 2.91	3.16 ± 5.59	0.846	2.88 ± 2.27	2.34 ± 1.34	0.294
Others	37.11 ± 16.70	64.85 ± 10.55	<0.0001	41.59 ± 13.99	67.05 ± 14.71	<0.0001	41.48 ± 15.31	69.61 ± 9.30	<0.0001

HTK, traditional Kochujang containing a high-dose of beneficial microbes; LTK, traditional Kochujang containing a low-dose of effective microbes; CK, commercially prepared Kochujang. Values are presented as the mean ± standard deviation. Comparison of microbiome changes in stools after Kochujang administration between three groups.

## Data Availability

Raw data can be provided upon request.
